# Stand-alone LLIF Lateral Cage Migration: A Case Report

**DOI:** 10.7759/cureus.347

**Published:** 2015-10-12

**Authors:** Wendy S Towers, Khalid H Kurtom

**Affiliations:** 1 Neurosurgery, University of Maryland Shore Regional Health

**Keywords:** cage migration, lateral lumbar fusion, minimally invasive spine surgery

## Abstract

Lateral approaches to the lumbar disc space have become popular in recent years with very few reported complications. We report on a rare case of a stand-alone cage migration.

A 77-year-old female presented with a right L2-3 radiculopathy that was refractory to maximum medical management. This was secondary to foraminal compression at L2-3 and L3-4 due to degenerative disc disease and levoscoliosis, as well as Grade 1 spondylolisthesis at both levels. A left-sided approach lateral lumbar interbody fusion was performed at L2-3 and L3-4 using a lordotic polyetheretherketone (PEEK) graft (50 mm length x 18 mm width x 9 mm height) packed with demineralized bone matrix (DBM). A contralateral release of the annulus fibrosis was performed during the decompression prior to graft insertion. Postoperative anteroposterior and lateral x-ray imaging confirmed good position of interbody grafts, correction of scoliosis as well as spondylolisthesis, and restoration of disc height achieving foraminal indirect decompression. A routine postoperative x-ray at three months demonstrated asymptomatic ipsilateral cage migration at the L2-3 level with evidence of arthrodesis in the disc space. This was managed conservatively without further surgical intervention.

Placement of a lateral plate or interbody intradiscal plating system in patients with scoliosis and significant coronal deformity is an option that can be considered to prevent this rare LLIF complication. Moreover, asymptomatic cage migration may be conservatively managed without reoperation.

## Introduction

The minimally invasive transpsoas lateral lumbar interbody fusion (LLIF) was first described by Ozgur, et al. [1] in 2006. Since then, it has become a valuable option for patients with degenerative disc disease, spondylolisthesis, foraminal stenosis, tumor, and trauma [1-4]. Stand-alone LLIF is a minimally invasive transpsoas approach to achieve indirect decompression of the neural elements while restoring disc height and spinal alignment without other fixation measures, such as pedicle screws or lateral plate. The LLIF permits thorough disc removal and preparation of the graft bed. Indirect decompression is achieved by laterally placing an interbody graft of polyetheretherketone (PEEK) while maintaining biomechanical ligamental structures, namely the anterior longitudinal ligament and annulus, both restricting motion [1]. The implant also spans the apophyseal ring, which avoids cancellous bone. and therefore, in theory, inhibits subsidence [5-6].

While avoiding major complications encountered by the anterior approach, such as injury to the retroperitoneal structures, great vessels and the sympathetic plexus, the LLIF has its own unique complications. The neural structures of the lumbosacral plexus can be injured [1]. Electromyogram (EMG) neuromonitoring is used to aid in the detection and avoidance of these neural structures. Sensory neural structures, such as the lateral femoral cutaneous nerve, cannot be identified with EMG; therefore, visual inspection of the surgical site is also important to avoid injuring this nerve [7]. A rare complication of the lateral interbody fusion is cage migration. It has been previously reported once in the literature, to our knowledge, in a patient that became symptomatic, necessitating a revision [8]. We report another case of stand-alone LLIF cage migration, in this instance, in an asymptomatic patient who was managed conservatively with observation.

## Case presentation

**Figure 1:**
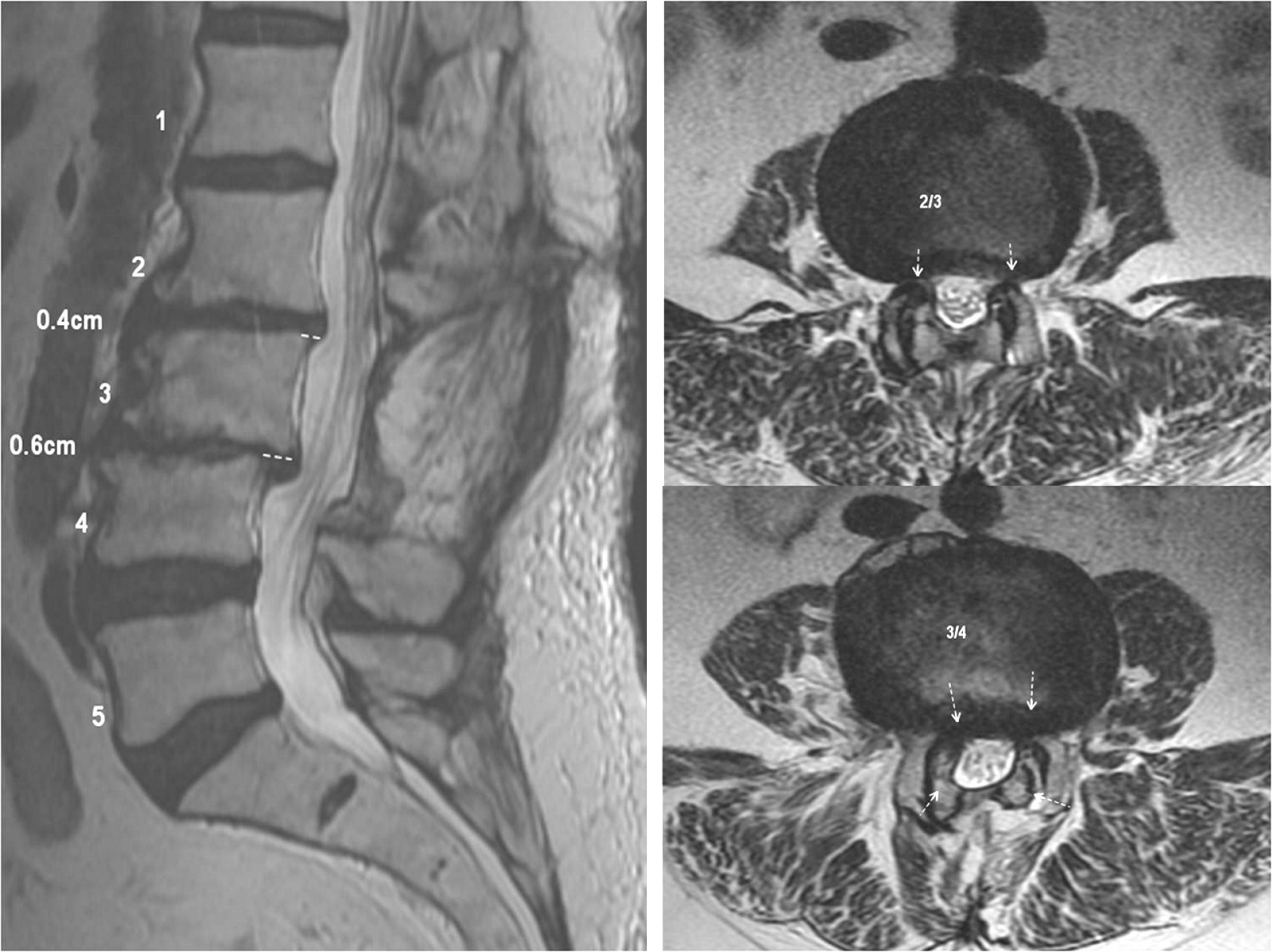
Preoperative MRI L2-3 and L3-4 degenerative disc disease, levoscoliosis, Grade 1 spondylolisthesis

**Figure 2:**
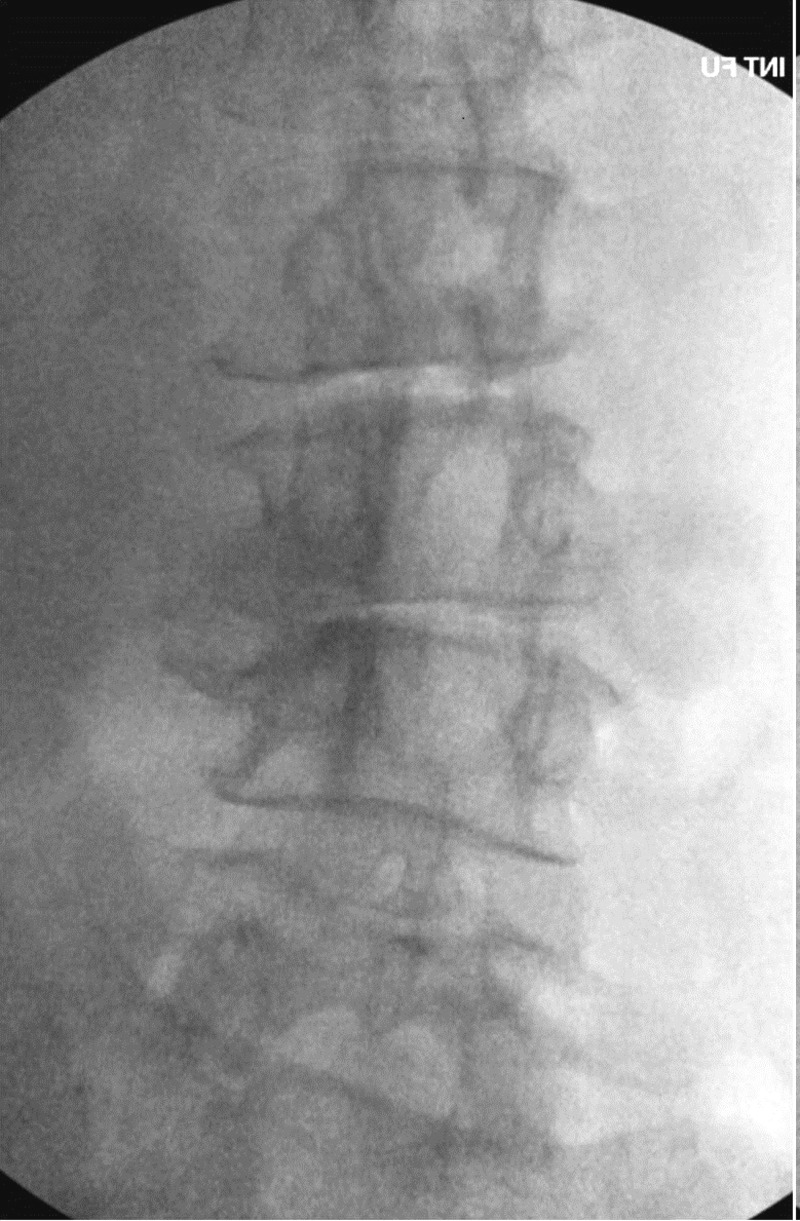
Preoperative x-ray Levoscoliosis L2-3, L3-4

Informed patient consent was obtained prior to treatment. With the patient’s permission, we report on a case of a 77-year-old female who presented with right L2-3 radiculopathy that was refractory to maximum medical management. This was secondary to foraminal compression at L2-3 and L3-4 due to degenerative disc disease, levoscoliosis, as well as grade 1 spondylolisthesis at both levels (Figures 1, 2).

**Figure 3:**
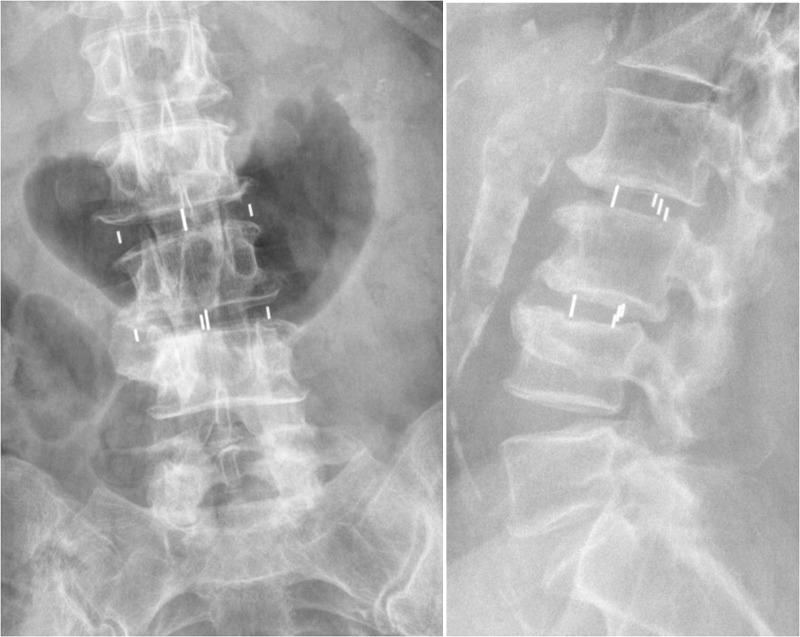
Immediate postoperative A/P and lateral fluoroscopy Cage position(s) in good A/P and lateral alignment with correction of scoliosis, spondylolisthesis, and restoration of disc height

Given the patient’s prior history of posterior decompressive laminectomy, it was felt that the best approach would be a minimally invasive lateral approach to obtain indirect decompression of the foramina as well as correct her spinal deformity. This will achieve the goals of decompression and fusion as well as deformity correction while avoiding the risk of operating through scar tissue. A retroperitoneal transpsoas approach was used to expose the disc spaces from the patient’s left side, initially at L3-4 followed by L2-3, using anteroposterior and lateral fluoroscopy imaging as well as neuromonitoring. Complete discectomy was performed with the release of the contralateral annulus fibrosis followed by the interbody fusion. A lordotic PEEK graft was used at each level, 50 mm length x 18 mm width x 9 mm height, packed with demineralized bone matrix (DBM). Postoperative anteroposterior and lateral x-ray imaging confirmed good position of interbody grafts, correction of scoliosis as well as spondylolisthesis, and restoration of disc height achieving satisfactory foraminal indirect decompression (Figure 3).

**Figure 4:**
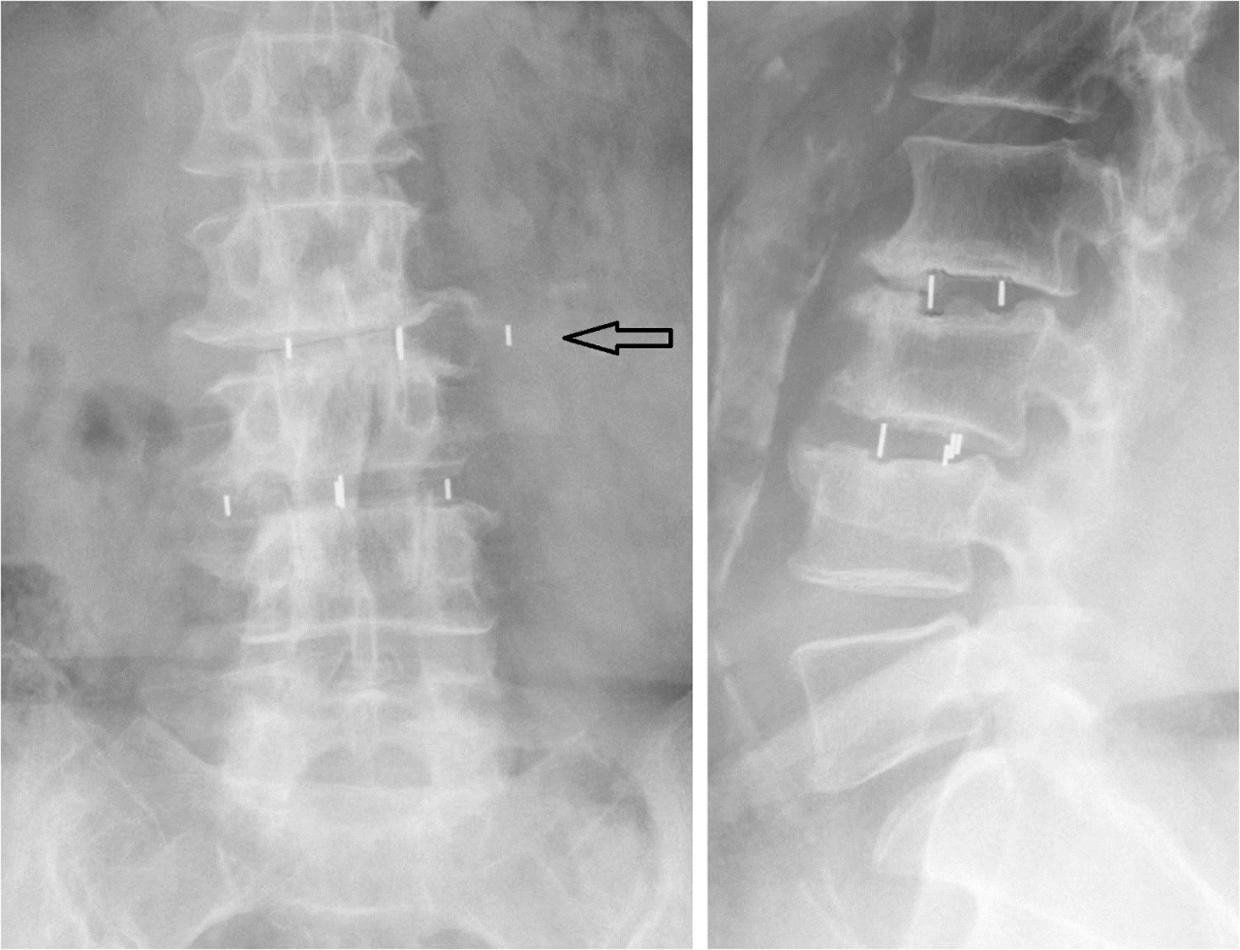
Three month postoperative x-ray Lateral cage migration at L2-3

A routine postoperative x-ray at three months demonstrated lateral cage migration on the left side at the L2-3 level with evidence of arthrodesis in the disc space (Figure 4).  

The patient was asymptomatic. Therefore, a decision was made to continue conservative management without surgical revision. She remained asymptomatic and follow-up imaging at one year demonstrated completed fusion without further migration of the interbody graft at L2-3.

## Discussion

Symptomatic adjacent level degeneration post lumbar spinal fusion occurs at a rate of 2%-3% per year as reported by Radcliff et al. [9]. Due to this rate of adjacent level disease progression and the increased risks associated with posterior re-operation, the lateral lumbar approach has become a valuable option especially for rostral adjacent segment degeneration above L4-5 [10]. Patients with degenerative spinal deformity or compression of the neural elements and prior posterior lumbar surgery also benefit from this approach with indirect decompression and restoration of normal spinal alignment.  The LLIF achieves anterolisthesis reduction, disc height restoration with foraminal decompression, and spinal fusion without complications associated with direct anterior fusion.

Minimally invasive LLIF, also referred to as extreme lateral interbody fusion (XLIF) or direct lateral interbody fusion (DLIF), can be performed as a stand-alone interbody fusion with posterior fixation or with lateral plate fixation. The anterior spine is approached through a retroperitoneal transpsoas incision as is described in various techniques with minor differences, hence, the procedure name variations [1, 8, 11-15]. The LLIF approach has its own unique complications. Technique directed complications are most often related to nerve injury as the retractor is placed through the psoas by a muscle-splitting technique [7, 16-18]. Neural complications are reported in 19-67% of patients and are described as anterior thigh pain, paresthesia, and dysesthesia as well as hip flexor weakness [19-21].

Femoral neuropraxia occurs most frequently when the level of spinal fusion is at the L4-5 disc space. Several studies have shown that the femoral nerve can lie directly over the center of the disc space at the L4-5 disc level, which increases its chance for injury; this can be devastating [22-25]. Grimm, et al. [16] suggested that limiting the retractor time at L4-5 may decrease the incidence of femoral nerve neuropraxia; however, they did not determine a safe timeframe. Davis, et al. [23] hypothesized that traction and compression of the femoral nerve against the L5 transverse process may not elicit a warning by neuromonitoring. Rodgers [18] determined that administering 10 mg of dexamethasone prior to incision for patients undergoing LLIF at the L4-5 level had zero neural deficits. Despite all attempts to reduce the risk of femoral neuropraxia during LLIF approach, this complication poses a concern for most surgeons performing this procedure.

A mode of failure of the implant in LLIF as well as other fusion techniques is subsidence. Le, et al. [26] studied 140 LLIF patients with a reported subsidence rate of 14.3% as demonstrated in anteroposterior and lateral radiographs. They defined subsidence as any compromise of the end plate. With this definition, they demonstrated a statistically significant positive correlation of subsidence with increasing construct length, with a 10.3% rate for one-level and up to 50% for four-level constructs. The rate of subsidence was only 1.9% with the 22 mm wide construct as opposed to 14.1% with the 18 mm wide construct. Of note, seven out 140 patients underwent stand-alone fusion with 0% subsidence in that group.

Our patient presented with scoliosis as well as spondylolisthesis, so an argument could be made to use supplemental posterior pedicle screw or lateral plate fixation in addition to the interbody cage. Marchi, et al. [2] studied 52 consecutive patients who underwent stand-alone LLIF for the treatment of low-grade spondylolisthesis with a 24-month follow-up. Postoperative complications were psoas weakness (19.2%), anterior thigh numbness (9.6%) that resolved within six weeks, and a 17% subsidence rate. There was no report of cage migration. Isaacs, et al. [17] reported perioperative outcomes and complications in a prospective, non-randomized multicenter study of extreme lateral interbody fusion for the treatment of adult degenerative scoliosis. They reviewed 107 cases with 18.7% patients receiving stand-alone XLIF. Of all the patients, 33.3% had some evidence of weakness after surgery with 6.5% not resolved within six months. They did not report subsidence rates. Complications did not include cage migration.

Lateral graft migration is a rare complication of LLIF, with a single reported case by Daffner and Wang [8]. They described a 50% laterally migrated interbody cage after an XLIF at L3-4 with supplemented posterior pedicle fixation. The patient was symptomatic. A revision was successfully completed with complete resolution of symptoms. They suspected that the maximal compression across the interbody graft was not achieved due to inadequate posterior forces. Another theory proposed is the contralateral annulus was inadequately released, and therefore, the residual coronal imbalance increased asymmetric pressure. The authors concluded a lateral plate should be utilized as reinforcement in patients that have a significant coronal abnormality or lateral listhesis adjacent to prior fusions in order to prevent graft migration. The proposed theories do not account for the complication in our patient since the contralateral annulus was fully released during the operation, resulting in the complete restoration of coronal imbalance post-graft placement. We do agree that a plating system could have prevented the complication encountered in our patient.

An intact anterior longitudinal ligament and posterior longitudinal ligament will prevent anterior-posterior graft migration, theoretically obviating the need for posterior fixation. Movement in flexion and extension should not destabilize the graft if both of these ligaments remain intact. On the other hand, proper technique during LLIF operation necessitates releasing both lateral annuli, so graft migration laterally is not restricted with lateral bending. This is remedied by placement of a lateral plate. Another option is to use an interbody with intradiscal plating system, which is especially useful in patients with prior fusion in adjacent levels.

## Conclusions

Lateral cage migration in LLIF is a rare complication. Asymptomatic lateral cage migrations can be conservatively managed without potentially risky revision procedures. Placement of a lateral plate or interbody intradiscal plating system in patients with scoliosis and significant coronal deformity is an option that can be considered to prevent this rare LLIF complication. 
